# Archaeosomes made of *Halorubrum tebenquichense *total polar lipids: a new source of adjuvancy

**DOI:** 10.1186/1472-6750-9-71

**Published:** 2009-08-13

**Authors:** Raul O Gonzalez, Leticia H Higa, Romina A Cutrullis, Marcos Bilen, Irma Morelli, Diana I Roncaglia, Ricardo S Corral, Maria Jose Morilla, Patricia B Petray, Eder L Romero

**Affiliations:** 1 Programa de Nanomedicinas, Universidad Nacional de Quilmes, Roque Saenz Peña 352, Bernal, Buenos Aires, Argentina; 2 Servicio de Parasitología y Enfermedad de Chagas. Hospital de Niños Dr. Ricardo Gutiérrez. Gallo 1330, Ciudad Autónoma de Buenos Aires, Argentina; 3 Laboratorio Ingeniería Genética, Biología Celular y Molecular- LIGBCM, Universidad Nacional de Quilmes, Roque Saenz Peña 352, Bernal, Buenos Aires, Argentina; 4 Centro de Investigación y Desarrollo en Fermentaciones Industriales-CINDEFI, Facultad de Ciencia Exactas, Universidad Nacional de La Plata, 50 y 115 La Plata, Buenos Aires, Argentina

## Abstract

**Background:**

Archaeosomes (ARC), vesicles prepared from total polar lipids (TPL) extracted from selected genera and species from the Archaea domain, elicit both antibody and cell-mediated immunity to the entrapped antigen, as well as efficient cross priming of exogenous antigens, evoking a profound memory response. Screening for unexplored Archaea genus as new sources of adjuvancy, here we report the presence of two new *Halorubrum tebenquichense *strains isolated from grey crystals (*GC*) and black mood (*BM*) strata from a littoral Argentinean Patagonia salt flat. Cytotoxicity, intracellular transit and immune response induced by two subcutaneous (sc) administrations (days 0 and 21) with BSA entrapped in ARC made of TPL either form *BM *(ARC-BM) and from *GC *(ARC-GC) at 2% w/w (BSA/lipids), to C3H/HeN mice (25 μg BSA, 1.3 mg of archaeal lipids per mouse) and boosted on day 180 with 25 μg of bare BSA, were determined.

**Results:**

DNA G+C content (59.5 and 61.7% mol *BM *and *GC*, respectively), 16S rDNA sequentiation, DNA-DNA hybridization, arbitrarily primed fingerprint assay and biochemical data confirmed that *BM *and *GC *isolates were two non-previously described strains of *H. tebenquichense*. Both multilamellar ARC mean size were 564 ± 22 nm, with -50 mV zeta-potential, and were not cytotoxic on Vero cells up to 1 mg/ml and up to 0.1 mg/ml of lipids on J-774 macrophages (XTT method). ARC inner aqueous content remained inside the phago-lysosomal system of J-774 cells beyond the first incubation hour at 37°C, as revealed by pyranine loaded in ARC. Upon subcutaneous immunization of C3H/HeN mice, BSA entrapped in ARC-BM or ARC-GC elicited a strong and sustained primary antibody response, as well as improved specific humoral immunity after boosting with the bare antigen. Both IgG1 and IgG2a enhanced antibody titers could be demonstrated in long-term (200 days) recall suggesting induction of a mixed Th1/Th2 response.

**Conclusion:**

We herein report the finding of new *H. tebenquichense *non alkaliphilic strains in Argentinean Patagonia together with the adjuvant properties of ARC after sc administration in mice. Our results indicate that archaeosomes prepared with TPL from these two strains could be successfully used as vaccine delivery vehicles.

## Background

In 1997, the pioneering work of Sprott showed that parenteral administration of nano-sized vesicles (archaeosomes, ARC) prepared with total polar lipids (TPL) extracted from microorganisms of the *Archaea *domain of life [1], produced a strong humoral response in mice [2]. Archaeal lipids exhibit radically different hydrocarbon backbones and polar head groups, as compared to polar lipids synthesized by organisms from *Eukarya *and *Bacteria *domains. Archaeal lipid backbones possess ether linkages and isoprenoid chains, mainly phytanyl and bysphythanediyl - archaeols and caldarchaeols- in *sn*-2,3 enantiomeric configuration, in contrast to the ester linkages, straight fatty acyl chains and *sn*-1,2 configuration of the glycerophospholipids from *Eukarya *and *Bacteria *domains [3]. Ether links are more resistant to acid hydrolysis than esters and the backbone/head group cross section of archaeal lipids is almost two folds higher than that of glycerophospholipids from *Eukarya *and *Bacteria *domains [4,5]. The same as liposomes, ARC can be prepared by self association of archaeal lipids upon a small input of energy in aqueous media (thin film hydration). However, beyond those apparent similarities, there are remarkable structural differences: the ARC surface is highly entropic, possessing half the surface tension than that of liposomes [5,6] and its permeability to protons and sodium cation is nearly one third of that determined for liposomes; the inclusion of macrocyclic archaeols and caldarchaeols further impairs ARC permeability to water and small solutes [7,8].

Those structural features make the ARC capable of establishing unique interactions with the biological environment, specifically eliciting adjuvancy to foreign proteins upon subcutaneous administration in preclinical models by strongly stimulating both the humoral as well as the cellular response, together with a sharp memory recall. Additionally, lipopolysaccharides are absent in *Archaea *[9] and, opposite to conventional immunomodulators that usually must be included into the liposomal structure [10,11], no toxicity has been found after parenteral administration of ARC, even at high or multiple dosage [12,13].

For strategic development of Third World countries, it is of crucial importance to count on vaccines of unproblematic storage-conservation, with high resistance to hydrolysis, oxidation and mechanical destruction, or easily reconstitutable upon lyophilization [14]. Adjuvants should preferably be biodegradable, non toxic, abundant, cheap and available from sustainable sources. Because ARC are suitable candidates to fulfill those requirements, it is relevant to survey the adjuvant properties of ARC made of TPL extracted from unexplored archaeal genera and species. In such context, we determined the cytotoxicity, intracellular transit and adjuvant activity of ARC prepared with total polar lipids of two *H. tebenquichense *strains isolated from Argentine Patagonia, upon two subcutaneous BSA doses followed by a single boosting inoculation in C3H/HeN mice.

## Results

### Strain isolation, growth and characterization

Halophilic archaea isolated from the upper gray crystals (*GC*) and the deeper black mud (*BM*) strata grown in enriched medium were characterized as disc-shaped (0.2 × 0.8 mm), motile Gram-negative microorganisms. Differentially, *GC *and *BM *colonies exhibited orange-red and reddish pigmentation, respectively. However, when grown in basal medium, a mixture of pleomorphic rods and disc-shaped motile archaebacteria displaying undefined Gram staining was observed.

The colonies grew in 10-20% NaCl-containing media, without Mg^+2 ^requirements, but optimum growth occurred at 20% NaCl, 40°C and pH 7.3 - 7.5. Remarkably, *GC *was able to grow at increased temperature (50°C). Both colonies were non-alkaliphilic, aerobic, oxidase and catalase positive. Only *GC *produced acid from a fructose source. Fructose, pyruvate, trehalose and galactose, but not starch, were used as sole carbon and energy sources by both colonies (Table 1). Both *GC *and *BM *organisms were chloramphenicol resistant and experienced no lysis even in the total absence of sodium salt.

**Table 1:** Phenotypic characteristics of the two colonies isolated from Argentinean Patagonia and the *Halorubrum tebenquichense *strain ALT6-92 isolated from Atacama saltern.

**Characteristics**	***BM***	***GC***	**ALT6-92**^a^
**Growth salt concentration (% w/v)**			

**10**	**+**	**+**	**-**

**15**	**+**	**+**	**+**

**20**	**+**	**+**	**nr**

**Growth at 50°C**	**-**	**+**	**+**

**Growth at pH 10**	**-**	**-**	**+**

**Cabalase**	**+**	**+**	**+**

**Oxidase**	**+**	**+**	**+**

**Acid from**			

**Xylose**	**-**	**-**	**-**

**Fructuose**	**-**	**+**	**-**

**Glucosa**	**-**	**-**	**-**

**Utilization of**			

**Fructose**	**+**	**+**	**+**

**Piruvate**	**+**	**+**	**+**

**Starch**	**-**	**-**	**+**

**Trehalose**	**+**	**+**	**+**

**Galactose**	**+**	**+**	**+**

**Hydrolysis of starch**	**-**	**-**	**-**

**G+C content (%)**	**59.5**	**61.7**	**63.2**

**% DNA-DNA similarity ^b^**	88.4 (94.0)	94.4 (91.9)	

^a ^Data obtained from Lizama et al., 2002

b Hybridization against *H. tebenquichense*. Values in parentheses are results of measurements in duplicate

Symbols: + positive result; - negative result; +/- slight mark; nr not reported.

For each isolate, a nearly complete 16S rDNA gene region (1300 bp) was sequenced (GenBank accession numbers GQ182977 and GQ182978) and compared with the same segment from other halophilic archaea. Phylogenetic analyses showed that both isolates belong to the Archaea domain and are related to the *Halorubrum *cluster, with the highest similarity to *Halorubrum tebenquichense *(98%). The G+C contents of the two isolates and the reference strain ALT-92T [15] were determined. *BM *and *GC *had a G+C content of 59.5 and 61.7 mol%, respectively (Table 1), whereas ALT-92T presented 63.2 mol%. On the basis of the *ad hoc *committee recommendations [16,17] of a threshold value of 70% DNA-DNA similarity for the definition of bacterial species, the two strains formed an homogeneous cluster with a high degree of internal similarity (DNA-DNA similarity > 90.5%) and should be considered as members of the same *H. tebenquichense *species.

**Figure 1:**
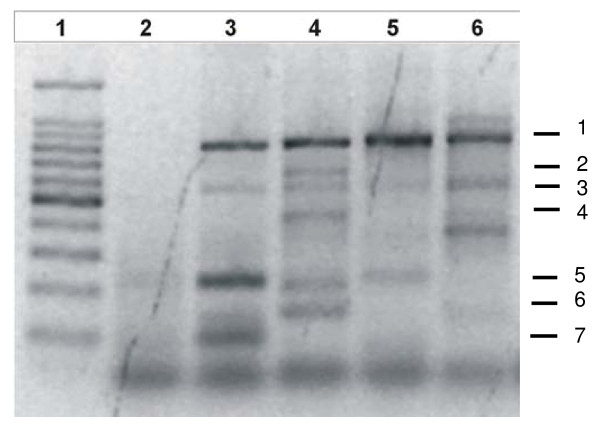
**AP-PCR finger print DNA analysis**. Lane 1: Ladder 100 pb -PB-L, lane 2: negative control, lane 3: *H. tebenquichense*, lane 4: BM, lane 5: GC, lane 6: *E. coli*.

The random amplified polymorphic DNA (RAPD) or arbitrarily primed polymerase chain reaction (AP-PCR) DNA fingerprinting technique provides one of the most sensitive and efficient of current methods for distinguishing different strains of a species [18]. In order to corroborate such differences between the two isolates, AP-PCR fingerprint analysis was performed (Figure 1). The band pattern showed visible differences between *BM *and *GC *fingerprint (absence or presence of 2, 4, 6 and 7 bands), in concordance with biochemical tests and 16S rDNA sequence data. In sum, differences in fructose metabolism, utilization of starch and optimum growth conditions (Table 1), together with those revealed by the AP-PCR, suggested that *BM *and *GC *isolates were different not only from those described for *H. tebenquichense *ALT-92 T [15], but also between each other and could be classified as new *H. tebenquichense *strains.

### Lipid extraction and characterization

The TPL, defined as the acetone insoluble portion of the total lipids, extracted from frozen cells obtained from a batch of 8 litres, ranged between 90 and 120 mg for each isolate.

A high content of phosphate groups is reported in the literature for the *Halorubrum *genus. Hence, for TPL quantification two colorimetric methods were applied: one, for detecting total phosphate (Bötcher) and the other for detecting organic phosphate (Stewart). Two calibration curves were prepared employing dry mass of TPL as standards that resulted linear between 10-40 μg for Bötcher and 20-400 μg for Stewart, with correlation coefficients exceeding 0.997, for each source of TPL.

On the other hand, calibration curves using inorganic phosphate (NaH_2_PO_4_) as standard were prepared with the aim of determining the phosphate percentage of archaeal lipids by the Bötcher method. A linear plot of dry mass of TPL vs. μg inorganic phosphate was determined and the relative amount of phosphate in the extracted TPL was similar for both isolates, in the order of 6.25% w/w.

### ESI -MS polar lipids profile

**Figure 2:**
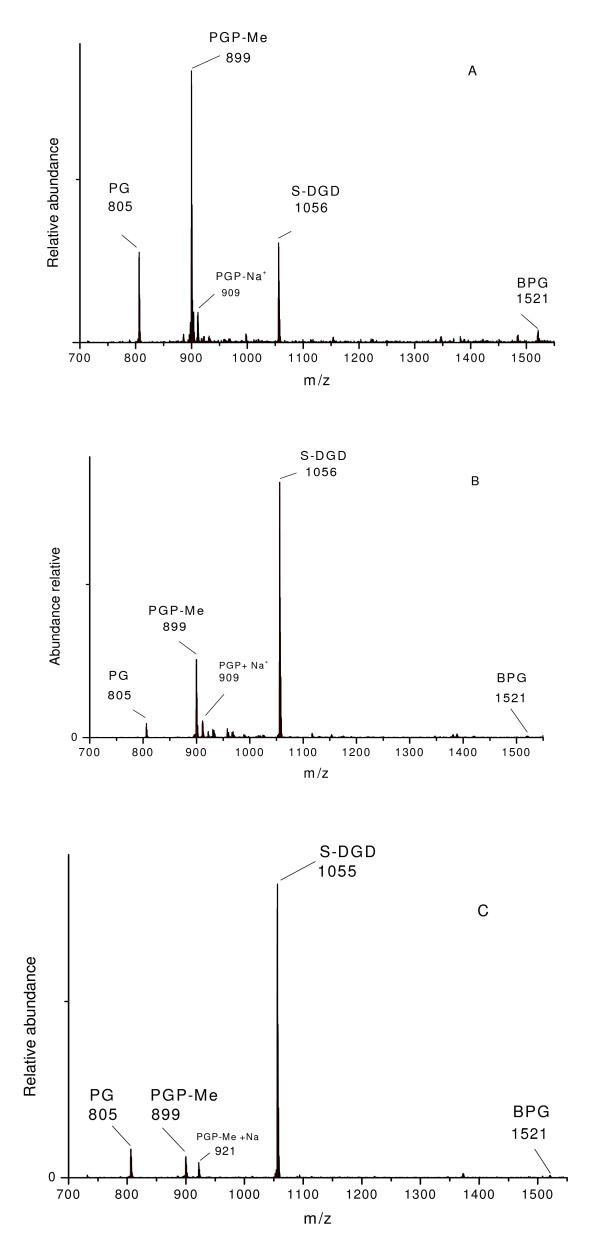
**Electrospray ionization-mass spectrometry (ESI-MS) analysis of polar lipids from A) *H. tebenquichense*, B) *BM *and C) *GC *extract**.

The ESI-MS spectrum (negative ions) analyses of the TPL of *H. tebenquichense*, *BM *and *GC *showed three main peaks at m/z 805, 899 and 1055.9 (Figure 2). In addition, *GC *displayed intense ion peak at m/z 1521 that was less intense for *H. tebenquichense *and *BM *extracts. The peaks at m/z 731.5 (diagnostic of PA) and 886 (PGS) that were reported for *Halorubrum sp*. [19] could not be detected in any of our samples. Negative ion ESI-MS (Figure 2A, B and 2C) identified major signals corresponding to PG m/z 805.7, PGP-Me m/z 899.5 (as monocharged peak), 449 (as bicharged peak) and S-DGD m/z 1055.7 (as monocharged peak). The diagnostic peak of archaeal cardiolipin (BPG) at m/z 760 (as bicharged peak) and 1521 (monocharged peak) was detected only as a small signal in the three TPL extracts.

### ARC characterization

As revealed by transmission electron microscopy, the two ARC preparations were multilamellar, with a mean size of 564 ± 22 nm and zeta-potential near to -50 mV. BSA incorporation did not modify size or zeta potential, the protein/lipid ratio was 20 μg/mg and the encapsulation efficiency around 3-4%.

### ARC uptake by cells and cellular toxicity

**Figure 3:**
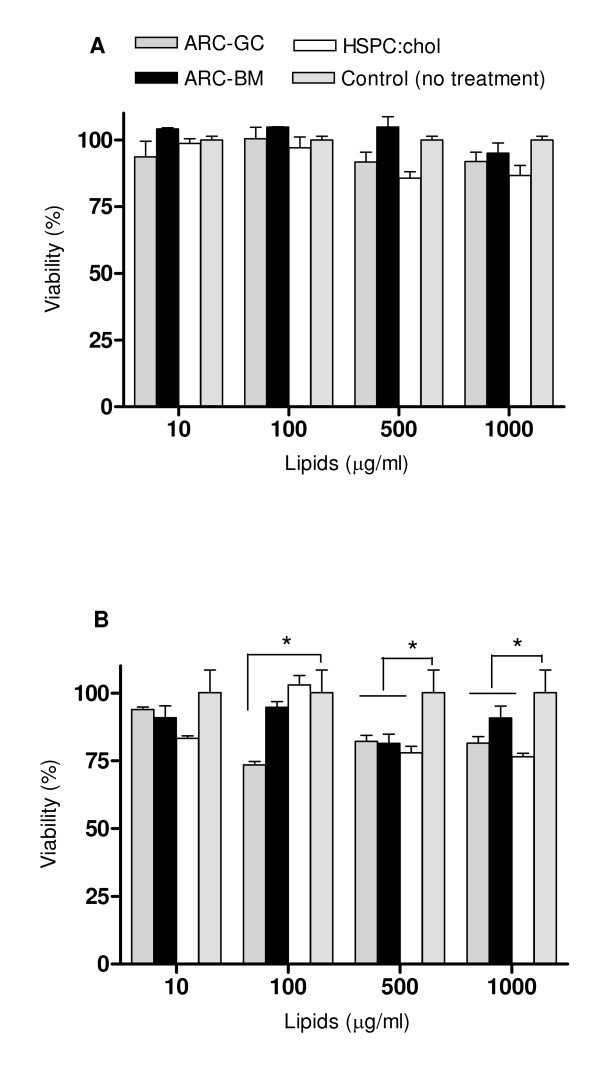
**Viability of Vero (A) and J-774 cells (B) upon 24 h incubation with ARC-GC, ARC-BM or HSPC:cholesterol liposomes, as function of concentration**. Values represent the average of triplicates ± S.D. ANOVA, * *p *< 0.05.

None of the ARC or HSPC:cholesterol liposomes significantly reduced the viability of non phagocytic cells (Vero cell line) upon 24 h incubation (Figure 3A). On the other hand, 10 μg/ml ARC-GC was non cytotoxic but the higher concentrations reduced cell viability by 25%, while increased concentrations (up to 100 μg/ml) of ARC-BM and HSPC:cholesterol liposomes did not affect cultured macrophages (J-774 cell line) (Figure 3B).

**Figure 4:**
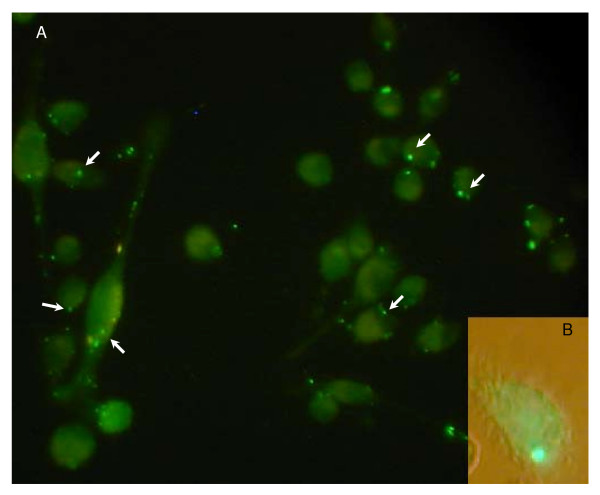
**(A) Fluorescence microscopy images of J-774 cells upon 30 min incubation with ARC-*BM*-HPS/DPX**. (B) Intracellular distribution of HPTS in green vesicles persistent 60 min after uptake.

As shown in Figure 4A, and independently of the TPL source, a minimal time period of 30 min was required for ARC uptake by phagocytic cells, as judged by the detection of intracellular fluorescence after incubation with both ARC-HPTS/DPX. In addition, J-774 macrophages incubated 45 min with both ARC- HPTS/DPX showed punctual fluorescence (as shown by arrows in Figure 4A) that resulted from the confinement of the HPTS/DPX into vesicular compartments in the cytoplasm. In case membrane fusion between ARC and the phagosome occurred, the ARC inner content should be released to the cytoplasm. Consequently, the HPTS would be dequenched from DPX and an homogeneous brightness filling the cytoplasm should be observed [20,21]. However, the punctual emission upon excitation at 440 nm, indicating confinement of the pair HPTS/DPX in acidic compartments, remained unchanged. Hence, the ARC did not fuse/disrupt, staying inside the phagosomes for at least 60 minutes (Figure 4B).

### Antibody response

**Figure 5:**
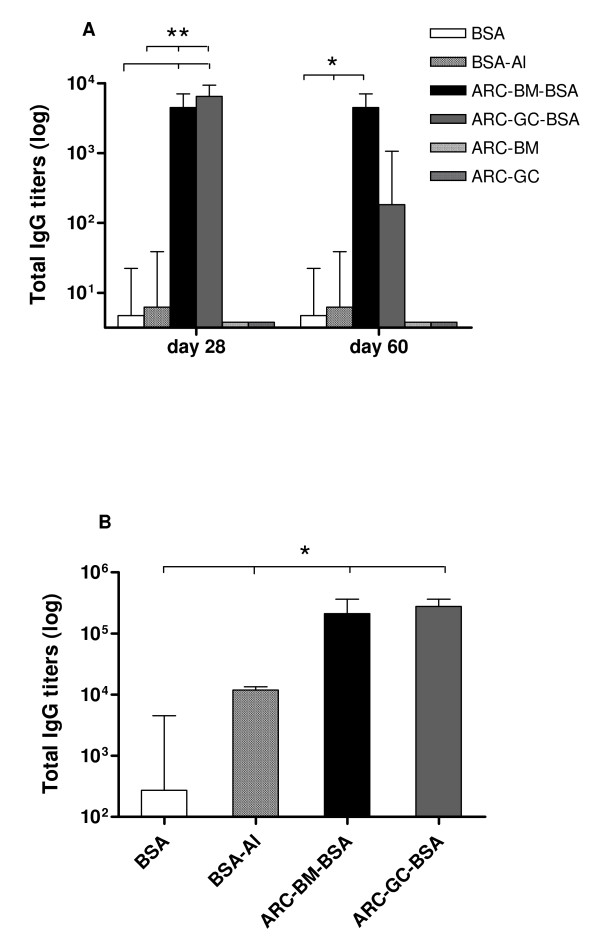
**Humoral response to ARC-entrapped BSA**. (A) Primary antibody response. Mice were immunized sc on days 0 and 21 with 25 μg of BSA alone, BSA adsorbed on Al_2_O_3_, BSA entrapped in TEB-BM and TEB-GC or empty TEB. Serum samples were collected on days 28 and 60, and ELISA assayed for anti-BSA antibody titers. (B) Long-term memory response to ARC-entrapped BSA. Mice were immunized sc on days 0 and 21 with 25 μg of BSA alone, BSA adsorbed on Al_2_O_3 _or BSA entrapped in ARC, and boosted on day 180 with 25 μg of BSA (without adjuvant). Serum samples were collected three weeks later and assayed for anti-BSA antibody titers. Values represent mean titers ± SEM. ANOVA, * *p *< 0.05, **p < 0.01.

To evaluate the adjuvant activity of both ARC formulations, we tested the antigen-specific humoral immune response after immunization of mice with BSA-loaded ARC. Following sc inoculations at 0 and 21 days, mice responded by day 28 with similar anti-BSA antibody (total IgG) titers for both ARC-*BM/GC*-BSA (Figure 5A), and presented a strong enhancement (~2 log, *p *< 0.01) of antibody titers over those of BSA and BSA-Al groups. Immunization with either empty ARC-*BM/GC *failed to evoke any anti-BSA IgG response, whereas only one out of four mice receiving adjuvant-free BSA displayed detectable specific antibodies. Mean titers in ARC-*BM/GC*-BSA groups were sustained until at least day 60 and declined by day 135. In all cases, very little anti-BSA IgM reactivity was detected throughout the study (data not shown).

To investigate the ability of ARC to generate long-term memory immunity, a boost-dose with BSA alone was injected on day 180. Three weeks later, a significantly higher serum antibody response (>1 log, *p *< 0.05) was detected in ARC-*BM/GC*-BSA compared with the BSA group (Figure 5B).

**Figure 6:**
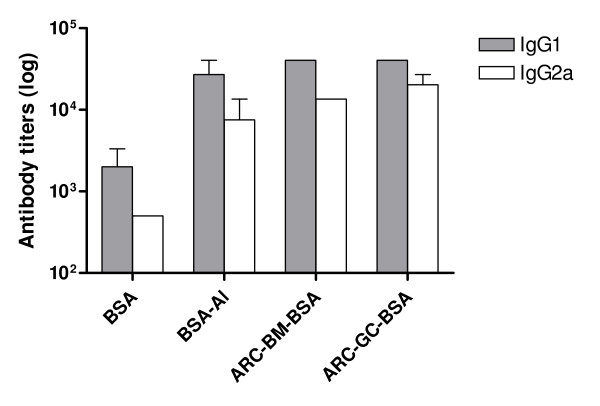
**Anti-BSA antibody isotypes induced after ARC-based immunization**. Mice were immunized as indicated in Fig. 5. Serum samples were collected three weeks after boosting and ELISA analyzed for IgG subclasses (IgG1 and IgG2a). Values represent mean titers ± SEM.

We further examined the IgG isotype distribution in the sera of immunized mice on day 200. Both IgG1 and IgG2a increased antibody titers could be demonstrated in long-term responses from ARC-*BM/GC*-BSA groups (Figure 6).

## Discussion

The extreme halophilic archaea genus *Halorubrum *is worldwide distributed [22-28] either at high altitude as well as under the sea level [15,25,29]. The only *Halorubrum *species so far described in South America is the *Halorubrum tebenquichense *ALT6-92 strain isolated from the water of Lake Tebenquiche at the unique environment of the Atacama saltern, located in northern Chile at 2300 metres above sea level [15]. Here we report the finding of new *H. tebenquichense *non alkaliphilic strains in a salt flat from the littoral of the Argentinean Patagonia, the Salina Chica in Península de Valdés (42° 32' S, 63° 59' W), at the province of Chubut. Remarkably, the Salina Chica is separated from Lake Tebenquiche by the highest (nearly 7000 m) and younger (70 million years) mountains in America, the Cordillera de los Andes, distant 2500 km south-east away and placed 40 meters below sea level in a zone of temperate weather.

The membranes of extremely halophilic archaea such as *H. tebenquichense *have several unique characteristics that vary little within specific genera [30]. Phospholipids with ethanolamine, inositol, and serine head groups are generally absent and specific phosphatidylglycerol phospholipids, sometimes including several sulfated glycolipids, predominate. Extreme halophiles contain 50-80% archaetidylglycerol methylphosphate (PGP-Me), an archaeal analogue of phosphatidylglycerol methylphosphate [31] that contributes to membrane stability in hypersaline environments [32], archaetidylglycerol (PG), and also some strains have minor amounts of sulfated PG [33].

Analysis of ESI-MS spectra of polar lipid isolates from *H. tebenquichense*, BM and GC, through comparison with the reference data [34,35] suggested the main phospholipids present in the extracts were phosphatidylglycerol (PG), phosphatidylglycerophosphate methyl ester (PGP-Me) and sulfated diglycosyl diphytanylglycerol diether (S-DGD). The prominent peak at 1055.9 corresponded to S-DGD and is representative of the *Halorubrum *genus [36]. The archaeal cardiolipin, bisphosphatidyl glycerol (BPG) was only found as traces in the three samples. While the complete lipid quantitation from each sample as well as the RMN identification of the S-DGD sulfonolipid will be accomplished in ongoing research.

Up to now, only ARC made of TPL extracted from *Methanobrevibacter smithii*, a methanogen archaea, are known to enhance the recruitment and activation of antigen presenting cells [37,38], induce co-stimulatory molecules expression [38], elicit CD8+ cytotoxic responses even in the absence of CD4+ helper T-lymphocytes [39], and to evoke a profound memory response, those all important stimuli that other vesicular systems such as liposomes, niosomes and ISCOMS are unable to trigger [40]. Additionally, although inflammation in innate responses can enable further adaptive responses [41], ARC made of TPL from *M. smithii *evoke immunologic memory in the lack of visible inflammation, activating dendritic cells in the absence of IL-12-dependent initial inflammatory response [42].

As regard to the extreme halophiles, ARC made of TPL extracted from *Halobacterium salinarum *was reported to induce a robust initial CTL and antibody response in mice [43]. Such titers, however, were not increased after boost administration and failed to induce a significant memory response. Only ARC made of TPL extracted from the hyperthermophile *Thermoplasma acidophilum *produced the most intense antibody memory responses to antigen challenge, together with a parallel induction of intense cell cycling in CD4^+ ^T cells suggesting efficient maintenance of T-cell memory [43]. A further study showed that among ARC from nine archaeal strains tested, all triggered remarkable primary CTL responses but only *M. smithii *and *T. acidophilum*, both rich in caldarchaeols, evoked a strong recall at > 50 weeks [44].

This is the first report of adjuvancy after parenteral administration of ARC-*BM/GC-*BSA. Both ARC elicited a strong and lasting primary antibody response, and remarkably in the absence of caldarchaeols in their lipid compositions, an enhanced memory humoral response after boosting with the bare antigen in C3H/HeN mice upon subcutaneous immunization. ARC-*BM/GC-*BSA initially triggered higher antibody titers than BSA formulated in alum adjuvant. Additionally, IgG isotype analysis of immunized mice revealed that both BSA-specific IgG1 and IgG2a antibodies were raised by both ARC-*BM/GC-*BSA, suggesting induction of a mixed Th1/Th2 response, in agreement with a previous report [39]. Our preliminary finding of humoral immune memory response elicited by ARC from extreme halophiles, together with established data on primary CTL and memory responses from ARC made of TPL from methanogens and hyperthermophiles, suggests that the headgroups are of crucial importance in the induction of immune responses, as recently determined by employing synthetic glycoarchaeols [45,46].

To sum up, even though our results for IgG isotyping in a single strain of mice (C3H/HeN) may not be extrapolated to the response in humans, it is promising to note that, in preclinical evaluation in animal model, ARC-*BM/GC*-BSA would appear as an efficient adjuvant delivery system to promote both humoral and, probably, cell-mediated immunity to the entrapped antigen.

## Conclusion

Two sc immunizations with either ARC-*BM/GC*-BSA, plus a boost with BSA alone rendered a long term humoral response stronger than that achieved with BSA formulated in alum.

Remarkably, our results were elicited in C3H/HeN mice, less prone to render potent humoral responses than BALB/c and C57BL/6 backgrounds [43]. Such preliminary results merit deeper insights on the search of CD8^+ ^CTL activity and the induction of this type of long term memory upon sc immunization with ARC. As judged by the phagosomal traffic followed by the pair HPTS/DPX loaded in ARC, our results indicated that there was neither fusion nor ARC content delivery to the cytoplasm, for at least 60 minutes post ARC uptake. Hence, cytoplasmic delivery of hydrosoluble material loaded in ARC-*BM/GC *should not happen, could either take longer than 60 minutes (considering that ARC-*BM*/*GC *were multilamellar, with negative zeta potential at physiological pH, both factors that impair or delay intermembrane fusion) or could occur through mechanisms other than the fusion mechanism recently reported for *Methanobrevibacter smithii *archaeosomes [47]. Finally, in spite of their TPL invariance, extreme halophilic archaea are source of glycolipid fractions that probably markedly influence the induction of primary responses and memory recall [30]. In view of that, the complete composition of ARC TPL and the mechanisms of recruitment, uptake and intracytoplasmic traffic of this antigen-delivery system are currently being analyzed in parallel with the ARC ability to induce expression of co-stimulatory molecules on professional APC.

## Methods

### Materials

Sodium 3'-[1-(phenylamino-carbonyl)-3,4-tetrazolium]-bis-(4-methoxy-6-nitro) benzene sulfonic acid hydrate (XTT), bovine serum albumin (BSA), antifoam 204 and cholesterol were provided by Sigma-Aldrich (Argentina). Hydrogenated phosphatidylcholine from soybean (HSPC) was obtained from Northern Lipids (Vancouver, Canada). RPMI 1640 culture medium was purchased from Invitrogen Corporation. Endotoxin-free fetal bovine serum (FBS) was bought from Hyclone. L-Glutamine, Trypsin, EDTA and penicillin/streptomycin were provided by PAA Laboratories GmbH (Austria). The fluorophore 8-hydroxypyrene-1,3,6-trisulfonic acid (HPTS) and the quencher *p*-xylene-bis-pyridinium bromide (DPX) were purchased from Molecular Probes (Eugene, OR, USA). *Halorubrum tebenquichense *strain ALT6-92 was purchased from Deutsche Sammlung von Mikroorganismen and Zellkulturen (DSMZ). Tris buffer and all the other analytical grade reagents were from Anedra (Argentina).

### Culture growth and characterization

Soil samples were collected from Salina Chica, Península de Valdés, Chubut, Argentina. Samples were classified according to their strata source as upper grey crystals (*GC*) and deeper black mud (*BM*). Isolation of microorganisms from each strata was done by seeding an aliquot on basal growth medium [48] with 1.6% agar (solid basal medium) at 37°C. The resulting colonies were grown on liquid medium at 40°C, at 160 rpm with chloramphenicol (30 mg/l) and then seeded on solid basal medium or brain-heart broth supplemented with yeast extract, glucose or blood (enriched medium).

Optimal NaCl concentration, pH, temperature and Mg^+2 ^requirements for growth were determined as described by Oren [49]. Gram staining as described by Dussault [50], cell shape and pigmentation examined by optical microscopy, were performed on colonies grown in basal medium and also in medium supplemented with blood or with brain-heart broth. Standard biochemical test methods were assessed for each colony.

Salt requirement for maintaining stability of the cell envelope was determined as described by Oren [49] by measuring the loss of turbidity of cell suspensions in medium of decreasing concentrations of NaCl (8, 6, 4, 2 and 0% w/v).

Biomass was generated in 8 l batch cultures in basal medium supplemented with yeast extract, glucose and antifoam (20 μl/l). Cultures were monitored by absorbance at 660 nm and harvested in late stationary phase for storage as frozen cell pastes.

### DNA isolation, G+C content

The DNA was isolated using a French pressure cell (Thermo Spectronic, Rochester, NY) and was purified by chromatography on hydroxyapatite as described [51]. The G+C content (mol%) of the DNA was determined by the midpoint value of the thermal denaturation profile (Tm) with a Perkin Elmer spectrophotometer at 260 nm, programmed for temperature increases of 1.0°C/min [52]. Tm was determined by the graphic method described by Ferragut and Leclerc [53] and the G+C content was calculated using Owen and Hill's equation (1979).

### PCR amplification of the 16S rDNA gene coding sequence and sequencing

Purified genomic DNA was used for PCR amplification of the16S rDNA gene. The following two sets of primers were used on the basis of the highly conserved regions of halobacterial 16S rDNA sequences as described by Ihara [48]: f1 (5' ATTCCGGTTGATCCTGC 3'), r1 (5' TTTAAGTTTCATCCTTG 3') and f2 (5' AACCGGATTAGATACCC 3'), r2 (5' GTGATCCAGCCGCAGATTCC 3'). PCR was performed with 35 cycles of 30 s at 94°C, 30 s at 37°C and 90 s at 72°C. The PCR products were analyzed by 1.5% agarose gel electrophoresis. The products were purified from the gel using S.N.A.P.™ Gel Purification Kit (Invitrogen) and sequenced directly by the dideoxy chain termination method [54].

### Phylogenetic analysis

The obtained sequences were compared with previously described 16S rDNA sequences of halophilic archaeas from the NCBI database. The sequences were aligned by using CLUSTAL X 1.83 [55,56] and phylogenetic trees were constructed by the neighbour-joining method with Kimura two-parameter calculation in MEGA 3.1 software. The confidence levels for branching orders were evaluated by the bootstrap method [57].

### DNA-DNA hybridization

DNA-DNA hybridization studies were performed as described by De Ley [58] under consideration of the modifications described by Huss [59] using a Cary^® ^100 Bio UV/VIS-spectrophotometer equipped with a Peltier-thermostatted 6 × 6 multicell changer and a temperature controller with in-situ temperature probe (Varian, Inc).

### DNA Fingerprint

DNA from the isolates was used for arbitrarily primed PCR (AP-PCR) fingerprint assay. Three small primers were used: T3GC (5' CCCAKTCGTGAWTCATGCT 3'), T3R, (5' TCCTCAYTTAATNAMCATGCT 3') and Rsh (5' ATCAAAAT 3'). PCR was performed for 35 cycles, starting with 10 s of denaturation at 92°C followed by 60 s of annealing at 30°C and 90 s of elongation at 72°C. The reaction mixture contained 10 ng of genomic DNA, 3 mM MgCl_2_, 1 μM of each primer, 0.2 μM of dNTPs, and 2 U of Taq Polymerase (Invitrogen) in a final volume of 20 μl. The PCR results were visualized in a 1.5% agarose gel electrophoresis and analyzed by Image Kodak Software.

### Lipid isolation

Lipids were extracted by the method of Bligh and Dyer as modified for extreme halophiles [60], from frozen and thawed biomass of each colony with Cl_3_CH:CH_3_OH:H_2_O (1:2:0.9; v:v), and the total polar lipid (TPL) fraction was collected by precipitation from cold acetone. Phospholipids of the TPL were quantified by Bötcher [61] and Stewart [62] methods.

### ESI-MS analysis

ESI-MS analysis was performed using a Thermo Finnigan LCQ Ion Max mass spectrometer (Thermo Finnigan MAT, San Jose, CA, USA) equipped with a electrospray ionization source. Analyses were carried out in the loop injection mode with dried lipid extracts dissolved in chloroform-methanol (1:1 vol/vol). Samples (5 μl) injected via a 10 μl loop were transferred to an MS electrospray interface (ESI) at flow rate of 10 μL/min. Interface conditions were as follows: nebulizer gas (air) 12 l/min, curtain gas (nitrogen), 1.2 l/min needle voltage - 5.0 kV (negative ions), mass range 50 - 2000 amu.

### ARC preparation and physicochemical characterization

Two different types of ARC were prepared from TPL isolated from each colony: ARC-*GC *and ARC-*BM*. Briefly, 20 mg of TPL from CHCl_3_: CH_3_OH (9:1, v/v) solution was rotary evaporated at 40°C in round bottom flask until organic solvent elimination. The thin lipid film was flushed with N_2 _and hydrated at 40°C with 1 ml of 10 mM Tris-HCl buffer plus 0.9% w/v NaCl, pH 7.4 (Tris buffer) (empty ARC) or with 1 ml of 10 mg/ml BSA solution in Tris buffer (ARC-*CG/BM*-BSA). The resultant suspensions were sonicated (20 min in a bath type sonicator 80 W, 40 KHz) and submitted to 5 cycles of freeze/thaw between - 80 and 37°C. Free BSA was removed by centrifugation and washing with Tris buffer (10000 × g for 20 min). Liposomes made of HSPC:cholesterol (1:1 mol:mol) were prepared in the same way.

The BSA/phospholipid weight ratio from each preparation was determined by phospholipid and protein quantitation. Phospholipids were quantified by Bötcher [61] whereas BSA content was measured by Bradford [63].

Mean ARC size was determined by dynamic light scattering with a 90 Plus Particle size analyzer (Brookhaven Instruments) and Zeta potential was determined with a Zetasizer 4 (Malvern). Electron microscopy images of ARC upon phosphotungstic acid negative staining were obtained with a TEM Jeon 1210, 120 Kv, equipped with EDS analyzer LINK QX 2000.

### Cytotoxicity

Upon incubation with ARC, cell viability was measured as mitochondrial dehydrogenase activity employing a tetrazolium salt (XTT) on Vero cells and the murine macrophage-like cell line J-774 [64].

Cells maintained at 37°C with 5% CO_2_, in RPMI 1640 medium supplemented with 10% heat-inactivated FBS, 2 mM glutamine, 100 UI/ml penicillin and 100 μg/ml streptomycin, were seeded at a density of 5 × 10^4 ^cells/well in 96-well flat bottom microplates. Culture medium of nearly confluent cell layers was replaced by 100 μl of medium containing 10, 100, 500 or 1000 μg/ml of lipids. Upon 24 h at 37°C, the cells were washed with 0.1 M phosphate buffered saline (PBS) pH 7.2, and incubated with 200 μg/ml of XTT for 4 h. XTT solution was removed and the extent of reduction of XTT to formazan within the cells was quantified by measuring the absorbance at 450 nm using an ELISA reader. Liposomes made of HSPC:cholesterol (1:1, mol:mol) were used as control.

### Cell uptake

Cell uptake and intracellular fate of ARC loaded with the fluorophore/quencher pair HPTS/DPX was followed upon incubation with J-774 macrophages by fluorescence microscopy. ARC loaded with HPTS/DPX was prepared as stated before, except that the lipid films were hydrated with a solution of 35 mM HPTS and 50 mM DPX in Tris buffer. J-774 cells grown to near confluence on rounded coverslips in 24-well plate were incubated with 10 μg/ml μg of ARC-*GC/BM*-HPTS/DPX at 37°C for 10, 20, 30, 45 and 60 min. After incubation, suspensions were removed, cells were washed, and coverslips were mounted on a fluorescence microscope. Cell-associated HPTS fluorescence was monitored using a Nikon Alphaphot 2 YS2 instrument.

Similarly, the intracellular fate of both fluorescent ARC upon 45 min-incubation with J-774 cells was followed by monitoring the HPTS fluorescent signal along 1 h.

### Immunization

Female 6-8-week-old C3H/HeN mice were obtained from University of Buenos Aires, Argentina, and maintained under standard conditions. The study was conducted following the Institutional Experimental Guidelines for Animal Studies. Groups of five animals were immunized subcutaneously (sc) on days 0 and 21 with 25 μg of BSA; 25 μg of BSA with added 100 μg of Al_2_O_3 _(BSA-Al, Alhydrogel^®^, Superfos Biosector, Vedbaek, Denmark) or 25 μg of BSA entrapped in any type of ARC (ARC-*GC*-BSA or ARC-*BM*-BSA; 1.3 mg of archaeal lipids each ARC). Control mice were injected with equivalent amount of empty TEB. All groups were boosted with 25 μg of adjuvant-free BSA on day 180.

### Evaluation of antibody response

Blood was collected from the tail vein at various time-points after immunization, as specified in the figure legends, and sera were analyzed by ELISA for the presence of anti-BSA antibodies. Briefly, microtiter plates (Nunc, Roskilde, Denmark) were coated overnight at 4°C with 45 μg/ml BSA diluted in 0.1 M carbonate-bicarbonate buffer (pH 9.6) and then blocked for 1 h at 37°C with PBS containing 0.2% Tween 20 (0.2% PBST) after washing with 0.05% PBST. Another wash as above described was followed by the addition of 100 μl of 3-fold dilutions of individual sera in 0.05% PBST. After 2 h at 37°C and further washing, the plates were incubated for 1 h at 37°C with horseradish peroxidase-conjugated goat anti-mouse IgG (Pierce, Rockford, IL) diluted 1:2000 in 0.025% PBST. For antibody isotyping, horseradish peroxidase-conjugated rat anti-mouse IgG1 or IgG2a revealing antisera (PharMingen, San Diego, CA), diluted 1:1000, were used. The plates were further washed and the reactions were developed by adding the ABTS substrate [2, 2'-azino-bis (3-ethylbenzthiazoline-6-sulphonic acid), Sigma Chemical Co., St. Louis, Mo]. Color was allowed to develop for approximately 10 min at room temperature in the dark. The optical density was measured at 405 nm using an ELISA reader (Multiskan Ex, Thermo Labsystems, Finland). Antibody titers are represented as end-point dilutions exhibiting an optical density of 0.3 units above background.

### Statistical analysis

Statistical analyses were carried out with the Prisma 4.0 Software (GraphPad, San Diego, CA, USA). Group means were evaluated by ANOVA with Tukey's analysis to compare individual groups. Values of *p *< 0.05 were considered significant.

## Authors' contributions

ROG performed the preparation and structural characterization of ARC (size and Z potential, electronic microscopy), as well as cytotoxicity, cell transit and immunization schemes. LHH grew the archaea strains, isolated the TPL and grew the J-774 and Vero cells. Also contributed to the cytotoxicity, cell transit and immunization schemes together with ROG. MB performed the DNA sequencing, C+G content and finger print analysis. IM performed the biochemical tests. RAC performed ELISA measurements. DIR performed the ESI-MS spectra and analysis. PBP and RSC designed and developed the immunization protocols, participated to the discussion of the results and manuscript preparation, and provided minor financial support. MJM coordinated the performance experiments and analysis of experimental results. ELR coordinated the experiments, wrote the manuscript and provided main financial support. All authors read and approved the final manuscript.
